# Novel Neurovascular Protective Agents: Effects of INV-155, INV-157, INV-159, and INV-161 versus Lipoic Acid and Captopril in a Rat Stroke Model

**DOI:** 10.1155/2012/319230

**Published:** 2012-01-04

**Authors:** Barry J. Connell, Bobby V. Khan, Desikan Rajagopal, Tarek M. Saleh

**Affiliations:** ^1^Department of Biomedical Sciences, University of Prince Edward Island, Charlottetown, PE, Canada C1A 4P3; ^2^InVasc Therapeutics, Atlanta, GA 30084, USA; ^3^Atlanta Vascular Research Foundation, 3562 Habersham at Northlake, Atlanta, GA 30084, USA

## Abstract

*Background*. Lipoic acid (LA), which has significant antioxidant properties, may also function as a potent neuroprotectant. The synthetic compounds INV-155, INV-157, INV-159, and INV-161 are physiochemical combinations of lipoic acid and captopril. We sought to determine if these compounds have neuroprotective potential following middle cerebral artery occlusion (MCAO) in rats. *Methods*. Male Sprague-Dawley rats were injected intravenously with captopril (1–50 mg/kg) 30 minutes prior to MCAO. Blood pressure, heart rate, baroreceptor reflex sensitivity, and infarct size were measured. In addition, dose response effect on infarct size and cardiovascular parameters was determined using INV-155, INV-157, INV-159, and INV-161 and compared to captopril and LA. *Results*. Pretreatment with captopril and LA at all doses tested was neuroprotective. The compounds INV-159 (0.5–10 mg/kg) and INV-161 (1–10 mg/kg) produced a significant,dose-dependent decrease in infarct size. In contrast, INV-155 and INV-157 had no effect on infarct size. *Conclusions*. Combined pretreatment with captopril potentiated the neuroprotective benefit observed following LA alone. Both INV-159 and INV-161 were also neuroprotective. These results suggest that patients taking combinations of captopril and LA, either as combination therapy or in the form of INV-159 or INV-161, may also benefit from significant protection against cerebral infarction.

## 1. Introduction

Hypertension prevalence is highly variable among populations worldwide. In the United States there is a disproportionate burden of this disease and its complications in African Americans [1]. African Americans have the highest prevalence of hypertension in the world, significantly higher than people of African origin living outside the United States [2]. According to the 2003-2004 National Health and Nutrition Examination Survey (NHANES) hypertension prevalence is 39.1% in African Americans and 28.5% in White Americans [3]. The increased prevalence of hypertension in African-Americans has been attributed to both genetic and environmental factors [4]. Additionally, hypertension is usually observed at a younger age in African-Americans and it results in more severe disease complications. This results in a significantly higher hypertension related mortality rate for African Americans, 49.9% and 40.6% for African American men and women, respectively, compared to 17.9% for the overall US population in 2004 [1].

Cardiometabolic syndrome, a constellation of common cardiovascular risk factors (obesity/overweight, atherogenic dyslipidemia, glucose intolerance, and elevated blood pressure) has been shown to directly increase atherosclerotic cardiovascular disease [5]. Of particular interest is the important role of the endothelium in vascular homeostasis. Endothelial changes are considered precursors to early changes in the atherosclerotic endothelium leading to chronic diseases including cardiometabolic syndrome. Patients are often prescribed antihypertensive drugs such as captopril in an attempt to lower their risk for cardiovascular disease [6]. In addition, patients diagnosed with this syndrome would have a high probability of endothelial dysfunction and/or biomarkers associated with these disorders. Anti-inflammatory and antioxidant therapies have been shown to reduce the biomarkers associated with this endothelial dysfunction [7]. In fact, in a cross-over, double-blinded study in patients diagnosed with cardiometabolic syndrome, our group has recently demonstrated that one such naturally occurring product *α*-lipoic acid (LA) improves endothelial function. This effect was potentiated in patients taking an angiotensin converting enzyme (ACE) inhibitor similar to captopril [7]. Since both captopril [8, 9] and LA [10] have been shown to provide neuroprotective effects, we designed the current study to determine if this combination therapy would also provide neuroprotection. The results provide support for our hypothesis that the combination of ACE inhibition and LA may provide additional cerebrovascular protection in patients with cardiometabolic syndrome.

## 2. Methods

All experiments were carried out in accordance with the guidelines of the Canadian Council on Animal Care and were approved by the University of Prince Edward Island's Animal Care Committee.

### 2.1. General Surgical Procedures

All experiments were conducted on male Sprague-Dawley rats (200–350 g; Charles River; Montreal, PQ, Canada). For all animals, food and tap water were available* ad libitum*. Rats were anaesthetized with sodium thiobutabarbital (Inactin; Sigma-Aldridge; St. Louis, MO, USA; 100 mg/kg; i.p.) which provided a stable plane of anesthesia for the full duration of the experimental time periods (no animals required anesthetic supplementation). To monitor blood pressure and heart rate, a polyethylene catheter (PE-50; Clay Adams, Parsippany, NJ, USA) was inserted into the right femoral artery. For intravenous administration of drugs a second polyethylene catheter (PE-10; Clay Adams, Parsippany, NJ, USA) was inserted into the right femoral vein. Arterial blood pressure was measured with a pressure transducer (Gould P23 ID, Cleveland, OH) connected to a Gould model 2200S polygraph. Heart rate was determined from the pulse pressure using a Gould tachograph (Biotach). These parameters were displayed and analyzed using PolyviewPro/32 data-acquisition and analysis software (Grass; Warwick, RI, USA). An endotracheal tube was inserted to facilitate breathing. Body temperature was monitored and maintained at 37 ± 1EC using a Physitemp feedback system (Physitemp Instruments; Clifton, NJ, USA).

### 2.2. Middle Cerebral Artery Occlusions

Our research group had previously published the detailed methodology for transient occlusion of the middle cerebral artery [11]. Briefly, animals were placed in a David Kopf stereotaxic frame (Tujunga, CA, USA) and the right middle cerebral artery (MCA) approached through a rostra-caudal incision of the skin and frontalis muscle at the approximate level of bregma. Blood flow through the MCA was impeded by the placement of surgical suture behind the MCA at 3 designated positions along the exposed vessel. The ends of the sutures were positioned so that the middle of each suture applied pressure to the MCA and completely impeded blood flow as confirmed using laser Doppler flowmetry (OxyFlo, Oxford-Optronix, Oxford, UK). This 3-point placement of surgical sutures produced a highly reproducible and consistent focal ischemic lesion restricted to the ipsilateral cerebral cortex. To facilitate removal of the sutures at the end of the occlusion period (30 minutes), a few drops of warm physiological saline (37EC) was first applied to the areas where the MCA was in contact with the sutures. Following removal of the sutures, blood was allowed to reperfuse the area for an additional 5.5 hours (I/R).

### 2.3. Cardiac Baroreflex Testing

To determine the effect of drug administration on the reflex bradycardia following baroreceptor activation, the baroreceptor reflex was evoked using a bolus intravenous injection of the *α*-adrenergic receptor agonist, phenylephrine-hydrochloride (Sigma-Aldridge; St.Louis, MO, USA; 0.1 mL; 2.5 *μ*g/mL; i.v.). The ratio of the peak change in the magnitude of the reflex bradycardia to the magnitude of the phenylephrine-induced pressor response (ΔHR/ΔMAP) was used as a measure of baroreceptor sensitivity [12] (BRS). BRS was tested 10 minutes and immediately prior to drug administration. BRS was then tested 15 minutes following drug administration (15 minutes prior to MCAO), and then 5, 10, and 20 minutes following MCAO, as well as immediately prior to suture removal at 30 minutes. The BRS was also tested 30, 60, 90, 150, 210, 270, and 330 minutes following suture removal.

### 2.4. Drug Development

The synthetic drugs INV-155, INV-157, INV-159, and INV-161 were obtained from InVasc Therapeutics Inc. (Atlanta, Georgia, USA). These are patented, proprietary drugs commercially available from InVasc Therapeutics Inc., and are sold for the treatment of cardiometabolic syndrome in humans. All of these compounds are based on the physiochemical synthetic combination of captopril and *α*-lipoic acid (LA) in a 1 : 1 ratio, but differ in their configuration (derivatives of the parent compounds).

### 2.5. Effect of Drug Administration on Infarct Volume, Blood Pressure, Heart Rate, and BRS

In the first experiment, to examine the effect of captopril on ischemic and reperfusion-induced cell death, administration of captopril (Sigma-Aldridge; St. Louis, MO, USA; 1, 5, 10, or 50 mg/kg; 1 mL/kg; i.v.; *n* = 4 to 7/group) or physiological saline (0.9% sodium chloride; 1 mL/kg; i.v.; *n* = 5) was made 30 minutes prior to the onset of MCAO. Thirty minutes following drug injection, the sutures were put in place for 30 minutes, followed by 5.5 hours of reperfusion. Infarct volume was measured following 5.5 hours of reperfusion and BRS was measured at the intervals described above.

In the second experiment, a concentration of captopril of 5 mg/kg was chosen as it did not produce a significant change in blood pressure or heart rate, and was coadministered in combination with various doses of lipoic acid (0.0005, 0.05 and 0.5 mg/kg, 1 mL/kg; i.v.; *n* = 6/group) which have been previously reported to have no significant effect on infarct volume in the same MCAO model developed in our laboratory [10]. This coadministration was done 30 minutes prior to MCAO. Following 5.5 hours of reperfusion, BRS were measured at the intervals described above and infarct volume was determined.

In the third experiment, we aimed to determine if administration of the combined drugs would result in a reduction in infarct volume. Therefore, captopril (5 mg/kg) and lipoic acid (0.5 mg/kg; 1 mL/kg; i.v.) were coadministered immediately prior to the removal of the sutures (30 minutes after MCAO) and infarct volume was measured following 5.5 hours of reperfusion.

In the fourth experiment, we compared the efficacy of the physiochemical combination of captopril-lipoic acid compounds (INV-155, INV-157, INV-159, and INV-161; 10 mg/kg; 1 mL/kg; i.v.; *n* = 4 or 5 per group) or vehicle (2% sodium bicarbonate; 1 mL/kg; i.v.; *n* = 4) administered 30 minutes prior to MCAO followed by 5.5 hours of reperfusion. INV-159 and INV-161 produced significant neuroprotection and were further studied to determine if the neuroprotection was dose dependent. In addition to 10 mg/kg, 0.1, 0.5, and 1 mg/kg (1 mL/kg; i.v.; *n* = 4/group) of INV-159 were administered and 0.1 and 1 mg/kg (1 mL/kg; i.v.; *n* = 4/group) of INV-161 were administered. Infarct volume and BRS was determined as described above.

### 2.6. Effect of Drug Administration on Blood Flow through the MCA

To examine the effect of systemic captopril, lipoic acid, captopril-lipoic acid combinations, INV-159, and INV-161 administration on cerebral blood flow, we used laser Doppler flowmetry to measure blood flow before, during and following MCAO. Laser Doppler signals from the MCA were recorded as relative values in blood perfusion units (bpu). The tip of a 0.5 mm probe (OxyFlo, Oxford-Optronix, Oxford, UK) was placed directly over the MCA just ventral to the bifurcation of the MCA to the frontal and parietal cortices. Warm physiological saline was applied to the area so that the probe was measuring blood flow through the saline. Blood flow through the MCA was measured at 10 and 5 minutes prior to drug administration, and then 5, 10, 15, 20, 30, and 60 minutes following drug administration.

### 2.7. Histological Procedures

At the end of each experiment (total of 6 hours for each rat), all animals were perfused transcardially with phosphate buffered saline (PBS; 0.1 M; 200 mls), the brains were removed and sliced into 1 mm coronal sections using a rat brain matrix (Harvard Apparatus; Holliston, MA, USA). Sections were incubated in a 2% solution of 2,3,5-triphenol tetrazolium chloride (TTC; Sigma-Aldrich; St. Louis; MO, USA) for 5 minutes. Infarct volumes for both sides of each brain section were calculated with the use of scanned digital images of each brain section and using a computer-assisted imaging system (Scion Corporation; Frederick, MD, USA). The sum total of all the individual infarct volumes provided the total infarct volume for each rat.

### 2.8. Statistical Analysis

Data were analyzed using a statistical software package (SigmaStat and SigmaPlot; Jandel Scientific, Tujunga, CA). All data are presented as a mean ± standard error of the mean (S.E.M.). Differences were considered statistically significant if *P* ≤ 0.05 by a one-way analysis of variance (ANOVA) followed by a Bonferroni post hoc analysis. When only two groups were being compared, Student's *t*-test was used.

## 3. Results

### 3.1. The Effect of Preadministration of Captopril on Infarct Volume

**Figure 1:**
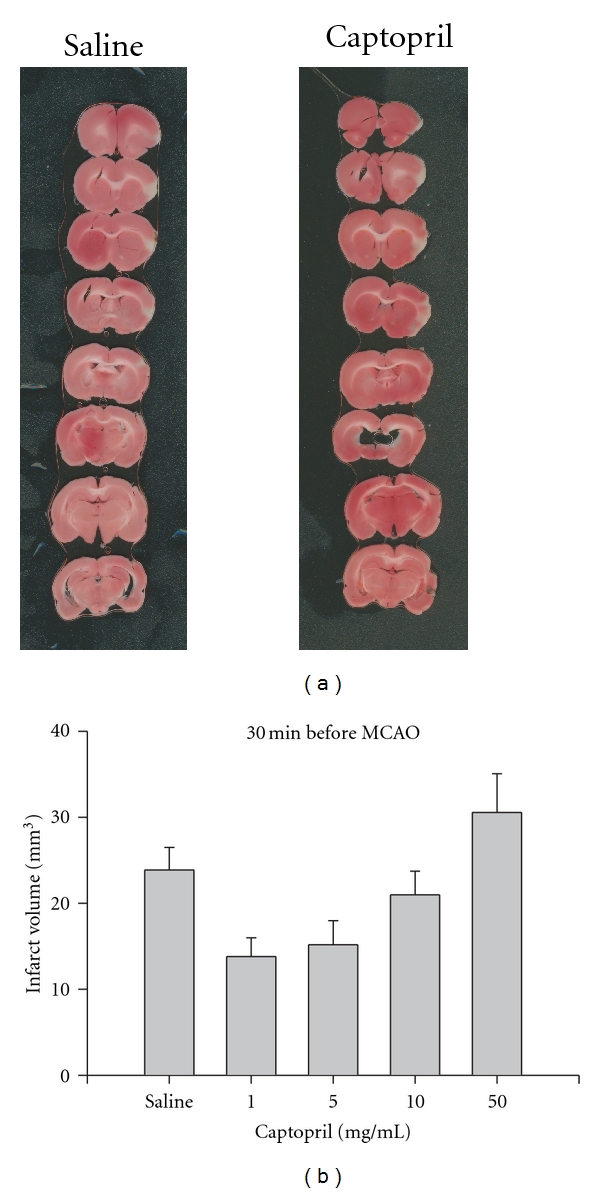
(a) Representative photomicrographs of TTC stained, 1 mm thick coronal slices illustrating the extent of the infarct within the prefrontal cortex following 30 minutes pretreatment (i.v.) with either saline or captopril (5.0 mg/kg) and ischemia/reperfusion (I/R). (b) Effect of pretreatment with either saline or captopril on infarct volume (mm^3^) calculated from TTC-stained 1 mm thick coronal sections throughout the extent of the infarct following I/R and pretreatment (i.v.; 30 minutes) with either saline or captopril. Each bar represents the mean ± S.E.M (*n* = 4–7/group).

The following experiment was designed to determine the effect of captopril pretreatment on MCAO-induced ischemia/reperfusion (I/R) injury. Captopril was administered 30 minutes prior to suture placement and infarct volume was measured following 5.5 hours of reperfusion. Captopril pre-treatment did not result in significant neuroprotection compared to saline with any of the doses tested (*P* ≥ 0.05; Figures 1(a) and 1(b)).

### 3.2. The Effect of Preadministration of the Combination of Captopril and Lipoic Acid on Infarct Volume

**Figure 2:**
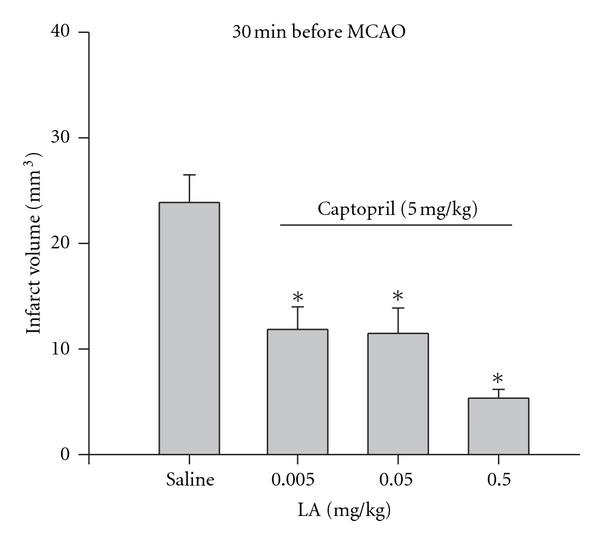
Effect of pretreatment (30 minutes before MCAO) with either saline (*n* = 5) or co-administration of captopril and lipoic acid (*n* = 6/group) on infarct volume (mm^3^) calculated from TTC-stained I mm thick coronal sections throughout the extent of the infarct following I/R. Each bar represents the mean ± S.E.M. and *indicates significance (*P* ≤ 0.05) from the saline control group (*n* = 4–7/group).

The following experiment was designed to determine the effect of coadministration of captopril with previously reported nonneuroprotective doses of lipoic acid on MCAO-induced ischemia/reperfusion (I/R) injury. The drug combinations were administered 30 minutes prior to suture placement and infarct volume was measured following 5.5 hours of reperfusion. Combining captopril (5 mg/kg) with each dose of lipoic acid (0.005, 0.05, and 0.5 mg/kg) resulted in significant neuroprotection compared to saline (*P* ≥ 0.05; Figure 2).

### 3.3. The Effect of Postadministration of the Drug Combination of Captopril and Lipoic Acid on Infarct Volume

**Figure 3:**
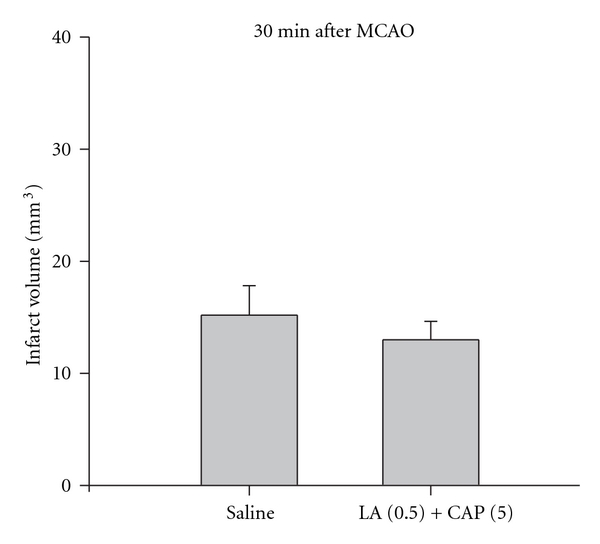
Average infarct volume (mm^3^) following either saline (*n* = 6) or a combination of captopril (5 mg/kg) and lipoic acid (0.5 mg/kg; *n* = 4) administration (i.v.) immediately prior to suture removal and the beginning of reperfusion (30 minutes after MCAO) calculated from TTC-stained coronal sections throughout the extent of the infarct. Each bar represents the mean ± S.E.M.

The following experiment was designed to determine the effect of the co-administration of captopril and lipoic acid on MCAO-induced ischemia/reperfusion (I/R) injury. Co-administration of the drug combination immediately prior to suture removal (30 minutes post-MCAO) did not result in a significant change in infarct volume compared to the administration of saline (*P* ≥ 0.05; Figure 3).

### 3.4. The Effect of Preadministration of the Compounds INV-155, INV-157, INV-159, and INV-161 on Infarct Volume

**Figure 4:**
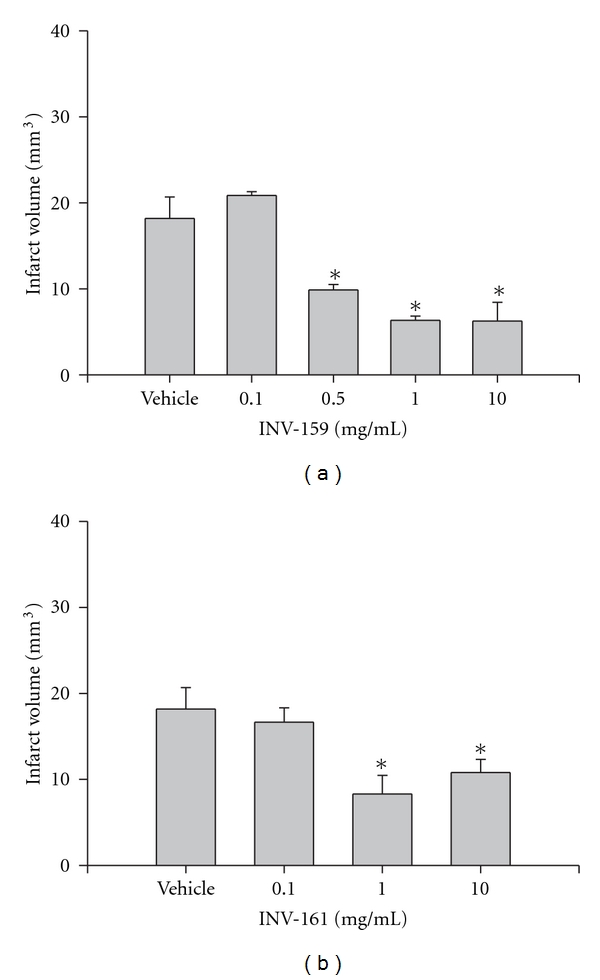
(a) Effect of pretreatment with either 2% sodium bicarbonate (vehicle), INV-159, or INV-161 (b) on infarct volume (mm^3^) calculated from TTC-stained 1 mm thick coronal sections throughout the extent of the infarct following I/R. Each bar represents the mean ± S.E.M. and *indicates significance (*P* ≤ 0.05) from the vehicle control group (*n* = 4-5/group).

The following experiment was designed to determine the neuroprotective capacity of the designer drugs INV-155, INV-157, INV-159, and INV-161 on MCAO-induced ischemia/reperfusion (I/R) injury. Each drug was administered 30 minutes prior to suture placement and infarct volume was measured following 5.5 hours of reperfusion. INV-155 (10 mg/kg) and INV-157 (10 mg/kg) did not result in significant neuroprotection compared to sodium bicarbonate vehicle (*P* ≥ 0.05; data not shown). However, INV-159 (10 mg/kg) and INV-161 (10 mg/kg) did result in significant neuroprotection compared to sodium bicarbonate vehicle and therefore complete dose-response curves were generated for each drug. INV-159 produced significant neuroprotection using doses of 0.5, 1.0, and 10 mg/kg (Figure 4(a); *P* ≤ 0.05 for each dose) and INV-161 produced significant neuroprotection using doses of 1.0, and 10 mg/kg (Figure 4(b); *P* ≤ 0.05 for each dose).

### 3.5. The Effect of Drug Preadministration on Cardiovascular Parameters

The following experiments were designed to determine the effect of drug preadministration on blood pressure, heart rate, and BRS before and following 30 minutes of MCA occlusion. Preadministration of captopril (1.0 mg/kg and 5 mg/kg) or saline did not significantly alter mean blood pressure or mean heart rate prior to, during, or following occlusion (*P* > 0.05; *n* = 4-5/dose; data not shown). However, the administration of captopril (10 and 50 mg/kg) resulted in a transient decrease in mean arterial pressure by  19 ± 5  and 34 ± 11 mmHg, respectively, (from an average baseline of 113 ± 9 mmHg) for ~5 minutes. This hypotensive effect was accompanied by a reflex tachycardia of  9 ± 2  and  19 ± 6  beats/min (from an average baseline heart rate of  412 ± 23  beats/min. When the BRS was tested following return of the cardiovascular parameters to baseline values 5 minutes after captopril injection, there was no significant difference compared to the preinjection value (0.55 ± 0.1 versus 0.5 ± 0.2).

Co-administration of captopril (5 mg/kg) with lipoic acid (doses 0.005, 0.05, and 0.5 mg/kg) 30 minutes prior to MCAO did not significantly alter mean arterial pressure, mean heart rate, or BRS prior to, during, or following occlusion compared to saline vehicle (*P* > 0.05; *n* = 5; data not shown). Administration of INV-159 (10 mg/kg) or INV-161 (10 mg/kg) did not significantly alter mean arterial pressure, mean heart rate, or BRS prior to, during, or following occlusion compared to vehicle (2% sodium bicarbonate; *P* > 0.05; *n* = 4/group; data not shown).

### 3.6. Effect of Drug Administration on Blood Flow through the MCA

To quantify blood flow through the MCA before, during, and following occlusion, we used laser Doppler flowmetry (Figure 4(b)). Administration of captopril (5 mg/kg), INV-159 (10 mg/kg) or INV-161 (10 mg/kg) 30 minutes prior to MCA occlusion did not result in significant changes in blood flow compared to preadministration values (*P* > 0.05; *n* = 4-5/drug; data not shown).

## 4. Discussion

The present investigation was conducted to determine if combination therapy using two known compounds, captopril and lipoic acid (LA) already prescribed and used by humans for the treatment of symptoms associated with cardiometabolic syndrome, could provide beneficial effects against cerebrovascular disease. The results of the present study supported our hypothesis that these drugs, in combination or synthetically combined, provide significant neuroprotection in a rat stroke model. In addition, no adverse cardiovascular complications were observed following pretreatment with any of these drugs, nor did they reverse the autonomic dysfunction observed following MCAO.

Cardiovascular and metabolic diseases (including atherosclerosis, stroke, and diabetes mellitus) represent the single largest global pharmaceutical opportunity within the pharmaceutical industry. The annual prescription drug revenue in this cardiometabolic space now exceed $400 billion annually. The advancing age of baby boomers, increased worldwide incidence of obesity, and a transition to a Western diet in much of the developing world clearly suggests that these chronic diseases will continue to be a primary driver within the industry. Cardiovascular and metabolic diseases represent huge product opportunities. However, the FDA have been driven by safety concerns and responding to congressional pressures associated with drug safety and efficacy benefit and the rising impact of healthcare on the gross domestic product. As a result, the agencies have become more restrictive in defining acceptable clinical endpoints. Furthermore, they are reducing the acceptance on surrogate endpoints in phase III trials designed for product approval. We believe that combining known nutraceuticals, such as LA, with approved prescription drugs (such as captopril) to magnify clinical benefits offer a significant development advantage and expedites the regulatory process for novel drug discovery.

In terms of the advantage of combination therapy, our results show that captopril significantly potentiated the neuroprotective effects of LA. Since captopril did not affect infarct volume or cerebral blood flow alone, it may not have facilitated access of LA to the brain. Therefore, the exact mechanism of this synergistic interaction requires further investigation.

In conclusion, our results support a role for the combination of captopril and LA to provide potential neuroprotection, and that this strategy of combination therapy for novel drug discovery should be further exploited.
